# Enhanced Photocharacteristics by Fermi Level Modulating in Sb_2_Te_3_/Bi_2_Se_3_ Topological Insulator p–n Junction

**DOI:** 10.1002/advs.202307509

**Published:** 2023-12-31

**Authors:** Seok‐Bo Hong, Dajung Kim, Jonghoon Kim, Jaehan Park, Seungwon Rho, Jaeseok Huh, Youngmin Lee, Kwangsik Jeong, Mann‐Ho Cho

**Affiliations:** ^1^ Department of Physics Yonsei University 50 Yonsei‐ro Seoul 03722 Republic of Korea; ^2^ Division of Physics and Semiconductor Science Dongguk University Seoul 04620 Republic of Korea; ^3^ Department of System Semiconductor Engineering Yonsei University 50 Yonsei‐ro Seoul 03722 Republic of Korea

**Keywords:** Bi_2_Se_3_, photocharacteristics, photodetector, Sb_2_Te_3_, topological insulator, topological insulator PN Junction

## Abstract

Topological insulators have recently received attention in optoelectronic devices because of their high mobility and broadband absorption resulting from their topological surface states. In particular, theoretical and experimental studies have emerged that can improve the spin generation efficiency in a topological insulator‐based p–n junction structure called a TPNJ, drawing attention in optospintronics. Recently, research on implementing the TPNJ structure is conducted; however, studies on the device characteristics of the TPNJ structure are still insufficient. In this study, the TPNJ structure is effectively implemented without intermixing by controlling the annealing temperature, and the photocharacteristics appearing in the TPNJ structure are investigated using a cross‐pattern that can compare the characteristics in a single device. Enhanced photo characteristics are observed for the TPNJ structure. An optical pump Terahertz probe and a physical property measurement system are used to confirm the cause of improved photoresponsivity. Consequently, the photocharacteristics are improved owing to the change in the absorption mechanism and surface transport channel caused by the Fermi level shift in the TPNJ structure.

## Graphical abstract

In TPNJ structures, variations in optical properties stem from the Fermi level approaching the surface bands, enhancing characteristics concurrently. This enhancement occurs when top and bottom independent surface channels are formed in topological insulators, Material's distinctive optical characteristics are profoundly influenced and delineated in the process.

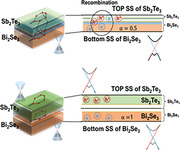

## 1 Introduction

Topological insulators (TIs), spotlighted as a future material owing to their unique phenomena, are being studied for applications in various fields such as optoelectronic devices,^[^ ^1^, ^2^ ^]^ quantum computing,^[^ ^3^, ^4^, ^5^ ^]^ and spintronics.^[^ ^6^, ^7^ ^]^ The high mobility, attributed to backscattering prohibition on topological surface state, and broadband absorption range due to the narrow band gap make TI excellent for applications as photodetectors.^[^ ^8^, ^9^, ^10^ ^]^ Excellent optical properties in broadband photodetectors using TI/Si heterostructure have been achieved.^[^ ^11^ ^]^ In particular, spin rectification known to increase spin current generation efficiency can occur in TI p–n junction (TPNJ) using different materials due to the spin‐momentum locking.^[^ ^12^ ^]^ Only vertically incident electrons with a specific spin direction pass through the p–n junction interface due to chiral tunneling and the Klein paradox.^[^ ^12^, ^13^ ^]^ When electrons are reflected from the interface, their spin is reversed due to spin‐momentum locking, resulting in a spin rectification effect. Many theoretical and experimental studies have been performed to implement the TPNJ structure owing to spin transportation for spintronics.^[^ ^14^, ^15^, ^16^ ^]^ To implement TPNJ structure in the vertical direction, growing different Tis by changing the composition of Bi and Sb has been reported.^[^ ^17^ ^]^ Moreover, forming TPNJ structure in the in‐plane direction was suggested by controlling the Fermi level using gating in a part of Bi_x_Sb_2‐x_Te_y_Se_3‐y_^[^ ^18^ ^]^ and deposition of the Sb bilayer on the Bi_2_Se_3_.^[^ ^16^ ^]^ These attempts have helped realize TPNJ with an interfacial band structure; however, electrical and optical properties characterized by the TPNJ structure based on the device were insufficient.

At the p–n junction interface, the built‐in field enhances the photogeneration and transportation of photocarriers. Similarly, the TPNJ structure is expected to have an advantage in terms of photocurrent generation efficiency. In addition, the surface state of the TI becomes a transport channel that suppresses scattering, contributing to the photocurrent generation efficiency. Recently reported optical properties based on TPNJ mainly showed improved photo characteristics by band bending between the two topological insulators; however, the cause of the improved optical properties in the TPNJ structure has not been investigated in detail.^[^ ^19^ ^]^ Directly extracting the cause of the properties in the photodetector is challenging owing to the various junction effects affecting the device characteristics, such as the optical process changed by the internal field as an internal factor and film condition including oxidation^[^ ^20^, ^21^ ^]^ as an external factor depending on environmental conditions.^[^ ^22^, ^23^ ^]^ Therefore, to explain the junction effect caused by intrinsic factors, the performance of devices manufactured under the same conditions needs to be compared. Because external factors can be excluded by comparing each characteristic in a single device through the proposed cross‐pattern design for the TPNJ, the internal junction effect affecting the device performance can be confirmed.

In this study, a TPNJ structure was optimized by controlling the growth temperature of Sb_2_Te_3_ on Bi_2_Se_3_ using an MBE system for enhanced interfacial coherency. Using X‐ray diffraction (XRD) and high‐resolution scanning transmission electron microscopy (STEM), the TPNJ structure was effectively implemented without intermixing. Furthermore, to confirm the junction effect in the p‐n structure, a cross pattern with a p–n junction was fabricated using stacked p‐ and n‐type TIs. By comparing the photocurrent characteristics of each p‐ and n‐type channel of the cross pattern with that of the junction channel of the stack structure with the same pattern, we successfully confirmed the photocurrent characteristics owing to the TPNJ structure. The effect of the surface state was investigated to clarify the cause of the improved photocurrent characteristics by analyzing the change in absorption and carrier dynamics related to the topological surface state depending on the Sb_2_Te_3_ thickness on Bi_2_Se_3_. Absorption spectroscopy, a physical property measurement system (PPMS), and optical pump‐THz probe spectroscopy (OPTP), revealed that with increasing Sb_2_Te_3_ thickness, the Fermi level of Sb_2_Te_3_/Bi_2_Se_3_ was modulated within the bulk bandgap, positioning it near the Dirac point of the surface state. Consequently, the increased absorption mediated by the surface state improved the optical properties. In addition, the surface channel of the TI enhanced photocurrent generation efficiency through increased transport efficiency of the separated carriers by suppressing backscattering and increasing the relaxation time owing to the Fermi level located in the bulk band gap. Thus, controlling the absorption and Fermi levels significantly enhanced the generated photocurrent.

## 2 Results and Discussion

**Figure 1:**
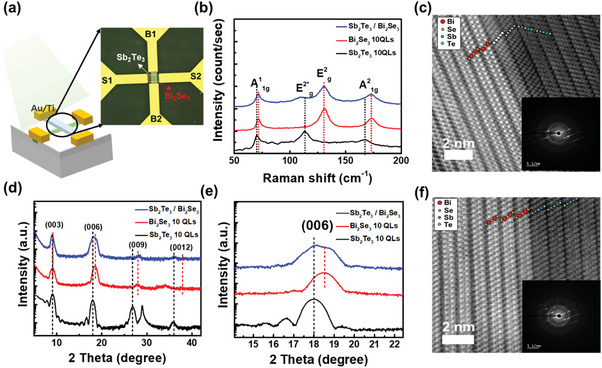
a) Optical microscopy image of the cross‐pattern device b) Raman spectroscopy results of Sb_2_Te_3_, Bi_2_Se_3,_ and c,f) Sb_2_Te_3_/Bi_2_Se_3_ STEM image of atomic resolution annealing temperature 180 and 170 ^°^C, respectively. d) X‐ray diffraction spectroscopy results of Sb_2_Te_3_, Bi_2_Se_3,_ and Sb_2_Te_3_/Bi_2_Se_3_ e) Graph plotted in log scale at (006) peak of (c).

Because various junction effects are included in the p–n junction, directly extracting the factors related to changes in the photo characteristics is challenging. A cross pattern was devised to confirm the photo characteristics of the TPNJ and the junction effects at the interfaces, excluding external influences, as shown in **Figure** **1**a. Using the cross pattern, depending on which source‐drain is connected, the characteristics of a single channel of p‐ or n‐type film and the p–n junction with the stack structure constituted by the two channels can be obtained under the same conditions. The device fabrication process shown in Figure S1 (Supporting Information) is explained in detail in the Methods section. Raman spectroscopy was conducted on each film and the stacked film, as shown in Figure 1b to investigate the changes in the state of the thin films during device fabrication. In Bi_2_Se_3_, three main peaks in the Raman spectrum corresponding to the A^1^_1g_, E^2^_g_, and A^2^_1g_ vibration modes at ≈70.8, 130.7, and 173.2 cm^−1^, respectively, were observed. For Sb_2_Te_3_, the main peaks of A^1^_1g_, E^2^_g_, and A^2^_1g_ vibration modes were observed at ≈69.9, 113.3, and 167.2 cm^−1^, respectively. These results on the characteristic Raman peaks are consistent with the previous studies for the epitaxially grown Bi_2_Se_3_ and Sb_2_Te_3_ films.^[^ ^24^, ^25^ ^]^ After growing Sb_2_Te_3_ on Bi_2_Se_3_ and annealing at 170 ^°^C, each Raman mode was observed distinctly. Regardless of the substrate type, such as SiO_2_/Si and Bi_2_Se_3_, a slight change in the E^2*^_g_ active mode of Sb_2_Te_3_ corresponding to the in‐plane vibration mode was observed.

XRD measurements were performed to confirm the epitaxial growth of the stacked films. As illustrated in XRD data of Figure 1d, films of Sb_2_Te_3_ and Bi_2_Se_3_ are epitaxially grown with *c*‐axis alignment; the stacked Sb_2_Te_3_ is also epitaxially grown on the Bi_2_Se_3_. The diffraction peaks observed in pairs were obtained from the stacked structure of Sb_2_Te_3_/Bi_2_Se_3_ in Figure 1e, indicating *c*‐axis aligned epitaxially grown Sb_2_Te_3_ and Bi_2_Se_3_ films. High‐resolution scanning transmission electron microscopy (STEM) measurements were performed on the two samples annealed at different temperatures to investigate the structural changes at the interface caused by the annealing conditions in detail. As shown in Figure 1c for the STEM data, since Bi appears relatively brighter owing to its larger atomic number than that of other atoms (Sb, Te, and Se), elements in the crystal structure can be distinguished using STEM technology. Moreover, this contrast in brightness makes it possible to distinguish a clear change in the crystal structure at the interface depending on the annealing temperature. At a relatively high annealing temperature (180 ^°^C), an intermixing layer is formed at the interface, whereas under appropriate annealing conditions (170 ^°^C), Bi_2_Se_3_ and Sb_2_Te_3_ grow with a distinct interface without any intermixing layer, as shown in Figure 1f. In addition, at 170 ^°^C annealing condition, Sb_2_Te_3_ grows in the same direction as Bi_2_Se_3_, whereas at 180 ^°^C annealing condition, Sb_2_Te_3_ is formed in a twin crystal structure due to a change in the lattice strain of the mixed layer. Through line profile analysis, two cases depending on the annealing temperature, namely, the interfacial layer at 180 ^°^C and abrupt interface without the interfacial layer at 170 ^°^C, were investigated, as shown in Figure S2 (Supporting Information).

To investigate the change in the formation of the intermixing layer at the interface depending on the annealing temperature, we identify changes in the chemical bonding state at the interface affecting the junction effect. The changes in the chemical bonding state at the 4interfaces, investigated using angle‐resolved X‐ray photoelectron spectroscopy (AR‐XPS), showed that no shift in the binding energy or new chemical bonding according to the angle change in the sample annealed at 170 ^°^C, as shown in Figure S3 (Supporting Information). In contrast, in the 180 ^°^C annealed sample, a shift in the binding energy of Bi and Se is observed at a low angle XPS data, as shown in Figure S4 (Supporting Information). This implies that a mixed layer of Bi atoms is formed and that the Se atoms move to the interfacial layer. The XRD, Raman spectroscopy, STEM, and AR‐XPS results confirmed that the TPNJ with the Sb_2_Te_3_/Bi_2_Se_3_ junction structure was effectively formed at 170 ^°^C to suppress intermixing.

**Figure 2:**
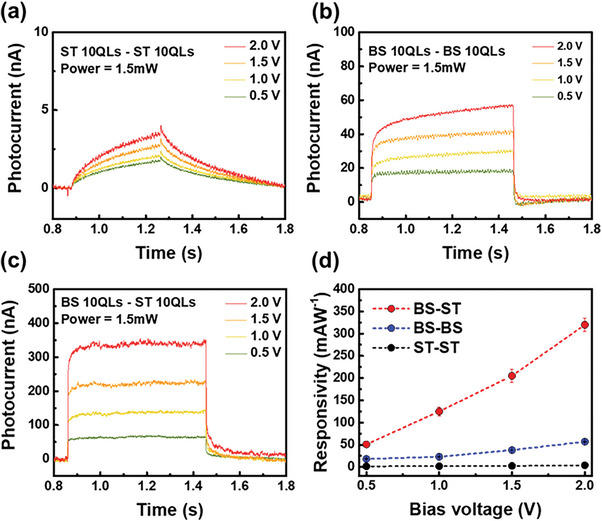
Photocharacteristics of TPNJ structure. Photocurrent results of a) Sb_2_Te_3_ (S1‐S2 contact) b) Bi_2_Se_3_ (B1‐B2 contact) c) Sb_2_Te_3_‐Bi_2_Se_3_ (S1‐B1 contact) d) Results of responsivity depending on bias voltage [0.5, 2 V].

Photocurrent measurements were performed while changing the metal lines connected to the source and drain of both channels in the cross‐pattern device to investigate the effect of the Bi_2_Se_3_/Sb_2_Te_3_ junction on the photodetector characteristics. The result of the measured photocurrent as a function of applied bias shows that photocurrent generation significantly depends on the channel type, as shown in **Figure** **2**a–c. When each source‐drain was connected to only Sb_2_Te_3_ (S1 and S2 in Figure 1a), the maximum photocurrent was 3.8 nA at a bias voltage of 2 V and a power of 1.5 mW. Sb_2_Te_3_ exhibits a slow response time compared to Bi_2_Se_3_ due to the strong thermo‐effect.^[^ ^26^ ^]^ When each source‐drain was connected to only Bi_2_Se_3_ (B1 and B2 in Figure 1a), the photocurrent increased to 56.25 nA for the same conditions. By connecting the source‐drain to S1 and B1 to observe the P–n junction effect, it can be seen in Figure 2c that the photocurrent intensity was significantly increased to 320 nA. To demonstrate the change in photo characteristics more clearly, we calculate the photoresponsivity using the photocurrent data, as shown in Figure 2d. The photoresponsivity *R* is defined as *R* = *I*_ph_/P, where I_ph_ is the intensity of the photocurrent and *P* is the applied power, representing the efficiency of photocurrent generation.^[^ ^27^ ^]^ The obtained photo‐responsivity value of Bi_2_Se_3_, Sb_2_Te_3_, and Bi_2_Se_3_‐Sb_2_Te_3_ were 3.8, 57, and 320 mAW^−1^, respectively. Because the photo characteristics were obtained for one device according to the contact position, the properties were reasonably compared under the same conditions without external factors for device fabrication. The nearly six‐fold improvement in photoreactivity over the Bi_2_Se_3_ *n*‐type and 84‐fold improvement over the Sb_2_Te_3_ *p*‐type indicate a strong effect on TPNJs.

To investigate the cause of the enhanced photocurrent characteristics in the TPNJ structure, which differed from the photocurrent intensity observed in Bi_2_Se_3_ and Sb_2_Te_3_, the change in the photocurrent was investigated by controlling the thickness of Sb_2_Te_3_ to adjust the Fermi level. Previous studies have shown that, as the thickness of Sb_2_Te_3_ increases, the Fermi level moves toward the valence band. In the case of Bi_2_Se_3_, the Fermi level exists in the conduction band owing to Se vacancy defects. Therefore, as the Sb_2_Te_3_ thickness grown on Bi_2_Se_3_ increased, the Fermi level could be adjusted toward the surface band by moving toward the valence band. This experiment allowed us to examine the contribution of surface states to photocurrent characteristics.

**Figure 3:**
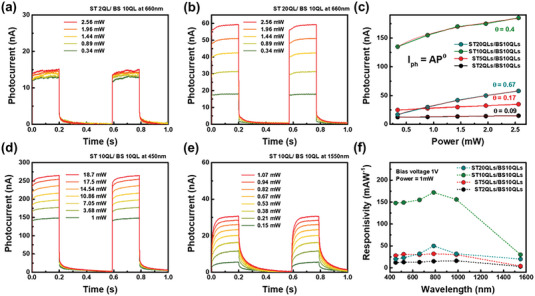
Photocharacteristics of TPNJ structure. Photocurrent results of a) Sb_2_Te_3_ 2 and b) 20 QLs grown on Bi_2_Se_3_ 10 QLs. c) Results of power law depending on the thickness of Sb_2_Te_3_^[^ ^2^, ^5^, ^10^, ^21^ ^]^ grown on Bi_2_Se_3_. Photocurrent results of Sb_2_Te_3_ 10 QLs/Bi_2_Se_3_ 10 QLs at wavelengths d) 450 and e) 1550 nm. f) Results of photo‐responsivity depend on the thickness of Sb_2_Te_3_^[^ ^2^, ^5^, ^10^, ^21^ ^]^ grown on Bi_2_Se_3_ at the wavelength range from 450 to 1550 nm.

In **Figure** **3**a,b, the maximum photocurrent increased from 15 to 60 nA at a power of 2.56 mW and a bias voltage of 1 V with a wavelength of 660 nm as the thickness of Sb_2_Te_3_ increased from 2 to 20 quintuple layers (QLs). Here, it is evident that the photocurrent intensity in the Sb_2_Te_3_ 2QLs/Bi_2_Se_3_ device in Figure 3a is lower compared to the BS‐BS channel in Figure 2b. For topological insulators, the band structure varies with thickness, and in the case of Sb_2_Te_3_ 2 QLs, the surface gap is open due to hybridization between the top and bottom surfaces.^[^ ^28^ ^]^ The decrease in photocurrent when surface bands are absent in Sb_2_Te_3_ 2 QLs implies that surface bands influence the photocurrent characteristics.

**Figure 4:**
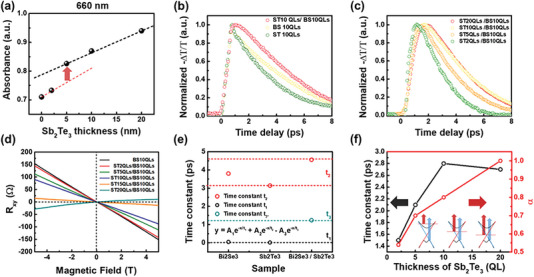
a) Changes in absorbance according to the thickness of Sb_2_Te_3_ at a fixed wavelength of 520 nm. Normalized differential amplitude −ΔT/T of the peak of the THz probe pulse was plotted for b) Sb_2_Te_3_, Bi_2_Se_3_, Sb_2_Te_3_/Bi_2_Se_3_ c) as the thickness of Sb_2_Te_3_ on Bi_2_Se_3_ increased from 0 to 20 QLs. d) Results of hall resistance with increasing Sb_2_Te_3_ thickness from 0 to 20 QLs. e,f) Time constant extracted from the results of b) and c), respectively. The decay time was fitted by $y\;=A_{1}\;e^{\frac{-x}{t_{1}}}+A_{2}e^{\frac{-x}{t_{2}}}$ for single thin films and $y\;=A_{2}\;e^{\frac{-x}{t_{2}}}-A_{3}e^{\frac{-x}{t_{3}}}$ for heterostructure.

In terms of absorption, the Sb_2_Te_3_/Bi_2_Se_3_ junction structure, where surface bands are merged, does not exhibit the influence of gap opening due to surface hybridization in Sb_2_Te_3_. However, in the case of Sb_2_Te_3_ with an open surface gap subjected to photo‐excitation, it strongly affects electron‐hole separation as carriers are transported through each film. This effect is observable through changes in the θ value (increased from 0.09 to 0.64 as shown in main Figure 3c). Further analysis on this aspect will be conducted later. To analyze the photocarrier generation efficiency in more detail, the power‐law equation I_ph_ = AP^θ^ was used, where *A* is the quantity associated with the responsivity, and *P* is the laser power. θ was obtained as a fitting parameter for comparison. The photocurrent results and changes in the photocarrier generation efficiency for each Sb_2_Te_3_ [2, 5, 10, 20 QLs]/Bi_2_Se_3_ composite at a wavelength of 660 nm as a power function are plotted in Figure 3c. As θ increases, the electron‐hole separation increases, thus increasing the photocarrier generation efficiency. The increase in θ with increasing Sb_2_Te_3_ thickness suggests efficient electron–hole separation occurs when the Fermi level is in the surface band. The reason for this phenomenon is explained in **Figure** **4**. Figure 3d,e show the photocurrent measurement results of the Sb_2_Te_3_10 QLs/Bi_2_Se_3_ 10 QLs films in response to lasers of wavelengths 450 to 1550 nm. As previously reported, topological insulators exhibit light absorption from the ultraviolet (UV) to infrared (IR) regions. This was confirmed by the photocurrent measurement results, indicating that the topological insulator was suitable for photodetectors. Figure 3f shows the responsivity plotted as a function of wavelength for each thickness. All the samples exhibited broad absorption characteristics. In particular, Sb_2_Te_3_/Bi_2_Se_3_ films with both 10 QLs show higher responsivity, indicating that the position of the Fermi level, which is determined by the thickness ratio of each film when fabricating a TPNJ structure, plays an important role in exhibiting a high light response in the surface band. Additionally, further analysis is needed to understand why the responsivity is higher for the Sb_2_Te_3_ 10 QLs sample, despite having a lower generation efficiency than the 20 QLs sample.

In general, the photocurrent generation efficiency is dependent on two factors: the absorption efficiency and the electron‐hole separation efficiency of the generated photoelectrons. In particular, θ in the equation for the photocurrent as the incident photon power function is closely related to the separation efficiency.^[^ ^29^, ^30^ ^]^ Therefore, the reason for the lower photoresponse in the Sb_2_Te_3_ 20 QLs/Bi_2_Se_3_ 10 QLs films, despite having a higher θ, is related to the change in absorption.

To investigate the effect of the Dirac surface on the absorption, we measured the absorbance depending on the thickness of Sb_2_Te_3_ grown on Bi_2_Se_3_, as shown in Figure 4a; the 660 nm wavelength used for photocurrent measurement was employed for measurement consistency. If Sb_2_Te_3_ only plays the role of an additional light‐absorbing channel to increase light absorption, the absorption should increase linearly according to Sb_2_Te_3_ thickness. However, at 5 QLs, the absorbance increased abruptly, as shown in Figure 4a, indicating that an additional effect occurs from a certain thickness. The data showed that the thickness was strongly related to the triggering of an abrupt increase in absorption. In the case of a topological insulator, the metallic surface state and the insulating bulk state affect absorption differently.^[^ ^31^, ^32^, ^33^ ^]^ In the bulk state, absorption bleaching (AB) occurs during the photoexcitation process: as the photo‐excited carriers in the bulk state occupy the empty conduction band, the empty state at the conduction band where the carriers can be excited is reduced and the absorption gradually decreases. However, in the case of photoexcited carriers generated in the surface state, free carrier absorption (FCA) can occur; already formed photoexcited carriers are excited to another unoccupied state in the same band, resulting in intraband absorption. That is, the abrupt increase in the absorption at 5 QLs can be closely related to FCA because of the generated TSS on 5 QLs and thicker Sb_2_Te_3_.^[^ ^34^ ^]^ Moreover, since the Fermi level can be controlled by the thickness of Sb_2_Te_3_,^[^ ^15^ ^]^ the absorption by the surface carriers increases as the film thickness increases.

According to a previous study using angle‐resolved photoemission spectroscopy while growing Sb_2_Te_3_ on Bi_2_Te_3_ can help modulate the Fermi level without changing the band structure.^[^ ^15^ ^]^ In other words, as Sb_2_Te_3_ grows on Bi_2_Se_3_, the Fermi level approaches the Dirac point, which changes the absorption mechanism. Hall resistance measurement, which can be performed under an applied magnetic field perpendicular to the direction of the current flow in the sample, makes it possible to determine the carrier type. The variation in the carrier type was confirmed by measuring the Hall resistance as a function of Sb_2_Te_3_ thickness on Bi_2_Se_3_ under a −5 to 5T magnetic field using PPMS. Thus, the variation in the position of the Fermi level can be confirmed.

The influence of the Fermi level was investigated using Figure 4d; as the thickness of the Sb_2_Te_3_ increases, the Fermi level at the interface is adjusted toward the valence band as the *p*‐type Sb_2_Te_3_ strongly influences it. Finally, at 20 QLs, the carrier type changed from *n*‐type to *p*‐type. Thus, the Fermi level can be modulated by increasing the thickness of Sb_2_Te_3_, resulting in the Fermi level moving near the Dirac point, which is a clear difference in the stacked structure, unlike the case where only Bi_2_Se_3_ is present. As the Fermi level approaches the Dirac point, backscattering is prohibited, and recombination is suppressed by reducing the scattering rate.^[^ ^35^, ^36^ ^]^ In our system, it is very difficult to measure changes in the Fermi level near the Dirac point using photoelectron spectroscopy because of measurement thickness limitations. Instead, the changes near the Dirac point confirm that the carrier relaxation time is reduced, and more carriers contribute to the FCA efficiency.

Understanding the carrier dynamics is important because changes in the absorption mechanism can be closely related to the carrier transition process. Since the carrier excitation and relaxation processes occur on the picosecond (ps) time scale in topological insulators,^[^ ^37^, ^38^ ^]^ optical‐pump terahertz‐probe (OPTP) spectroscopy with femtosecond time resolution can be used to investigate carrier excitation and relaxation. In the OPTP method, the carrier relaxation process can be observed by analyzing the transmittance change. The dynamic behaviors of the carriers extracted from the OPTP data of the Sb_2_Te_3_ 10  QLs, Bi_2_Se_3_ 10 QLs, and Sb_2_Te_3_ 10 QLs/Bi_2_Se_3_ 10 QLs films were compared. As shown in Figure 4c, the decay process in the stack structure of Sb_2_Te_3_ grown on Bi_2_Se_3_ occurs slowly as the Sb_2_Te_3_ thickness increases, indicating that phonon scattering decreases and relaxation slows as the Fermi level approaches the Dirac point. This phenomenon, occurring primarily in the intra‐band, varies within the picosecond time scale,^[^ ^36^ ^]^ consistent with Figure 4b,c. The decay spectra were fitted to investigate the carrier relaxation process after data normalization for a more detailed analysis. In the case of a film with a single layer, such as Sb_2_Te_3_ or Bi_2_Se_3_, the OPTP data are analyzed by $y\;=A_{1}\;e^{\frac{-x}{t_{1}}}+A_{2}e^{\frac{-x}{t_{2}}}$, where *t*_1_ is the recombination time at a channel that proceeds rapidly (intra‐ and inter‐valley recombination), and *t*_2_ is another recombination time at a channel that proceeds relatively slowly (inter‐band recombination).^[^ ^39^ ^]^ However, in the TPNJ structure with a stacked Sb_2_Te_3_/Bi_2_Se_3_ structure, the relaxation process can occur differently than that in a single layer. Since the gradual decline observed at ≈1 ps in the TPNJ structure of Figure 4b is caused by the negative components,^[^ ^40^, ^41^ ^]^ it is fitted by$\;y\;=A_{2}\;e^{\frac{-x}{t_{2}}}-A_{3}e^{\frac{-x}{t_{3}}}$(where A_1_ is zero). However, the change in relaxation within ≈1 ps containing both effects from the interfacial PN structure and Dirac surface states at both ends of Sb_2_Te_3_/Bi_2_Se_3_ cannot be distinguished. As the carrier density increases, the THz transmission decreases because of the inhibition of photocarrier interaction. That is, the first component with a negative amplitude contributes to the relaxation process as the THz transmission is increased after photoexcitation. As previously described for the absorption mechanism, intra‐valley scattering is decreased by tuning the Fermi level near the Dirac point, reducing recombination. Although the surface carrier on the Dirac surface state cannot be distinguished from the interfacial band structure, the decrease in intra‐valley scattering owing to the tuning of the Fermi level near the Dirac point is observed in Figures 4b,c. Moreover, the increase in rising time becomes slower, as shown in Figure 4c, because the FCA additionally contributes after filling the empty states.

The increased absorption efficiency of ≈10% cannot explain the nearly six‐fold change in the photocurrent. We noted that suppressing backscattering through the surface transport channel could also contribute to the photocurrent intensity.^[^ ^42^ ^]^ To explore the influence of the Dirac surface state in topological insulators, magnetoresistance (MR) measurements were conducted using PPMS. The Dirac surface state formation is attributed to the strong spin‐orbit coupling and weak antilocalization of the topological insulator. Additionally, the Hikami‐Larkin‐Nagaoka equation^[^ ^43^ ^]^ was employed to investigate the surface transport channel, dependent on the prefactor α value.

The Hikami‐Larkin‐Nagaoka (HLN) equation that relates the change of magneto‐conductivity (∆σ(𝐻)) to various physical parameters is expressed as follows:

(1) $$\Delta \sigma (H)\;=\sigma (H)-\sigma (0)\;=\;\frac{\alpha e^{2}}{\pi h}\left[\ln\left(\frac{B\phi }{H}\right)-\;\Psi \left(\frac{1}{2}+\frac{B\phi }{H}\right)\right]$$

where Ψ is the digamma function, e is the electronic charge, and h is Planck's constant. The characteristic magnetic field is represented by 𝐵𝜑, which is determined by the phase coherence length (𝑙_𝜑_) and coefficient (*α*) that characterize the localization type. The applied magnetic field is denoted as H. In topological insulators, *α* is related to the surface channel and equals 0.5 when there is a single surface channel. The fitting process was conducted within the magnetic field range of −0.3T to 0.3T. The detailed explanation is provided in the supporting information. In Bi_2_Se_3_ 10 QLs films, *α* = 0.72, which means that the top and bottom surface channels of Bi_2_Se_3_ are partially hybridized, resulting in two surface channels contributing to the transport process through the surface channels.^[^ ^44^ ^]^ The *α* value of Sb_2_Te_3_ 5 QLs films grown on Bi_2_Se_3_ is 0.54 because the hybridization effect can be generated between the bottom surface of Bi_2_Se_3_ and two surfaces of Sb_2_Te_3_. Furthermore, by increasing the thickness of the Sb_2_Te_3_, the top surface of Sb_2_Te_3_ can appear since the hybridization effect between the two layers decreases. Because there is no topological difference at the interface between Bi_2_Se_3_ and Sb_2_Te_3_, a Dirac surface state does not exist at these interfaces. The decoupling of surface states occurs at both ends between Bi_2_Se_3_ and Sb_2_Te_3_ as the thickness of the Sb_2_Te_3_ film changes, resulting in varying *α* values. In the case of 5 QLs Sb_2_Te_3_ + 10 QLs Bi_2_Se_3_, the interface where surface states can potentially form is located between the top surface of Sb_2_Te_3_ (between the vacuum and Sb_2_Te_3_) and the bottom surface of Bi_2_Se_3_ (between Bi_2_Se_3_ and the substrate). The HLN equation allows us to determine the number of surface channels from the *ɑ* value, with each *ɑ* value of 0.5 corresponding to one surface channel. Ideally, the ɑ value should indeed be 1. However, when measured using a Physical Property Measurement System (PPMS), *ɑ* values of 0.7 for Sb_2_Te_3_ 5 QLs, 0.8 for Sb_2_Te_3_ 10 QLs, and 1 for Sb_2_Te_3_ 20 QLs are observed, respectively. This indicates that the surface channels of Sb_2_Te_3_ and the surface states of the bottom Bi_2_Se_3_ are interacting with each other to some extent. When the top‐bottom surface states are not coupled, recombination can be suppressed because electrons are generated in Bi_2_Se_3_ and holes are generated during Sb_2_Te_3_ transport through separate channels. The PPMS and OPTP results show modulated Fermi levels near the bulk bandgap, increasing the absorption contribution of the surface carriers. Moreover, slowing the relaxation time and transport of the photoexcited carriers through the surface channel improves the photocurrent generation efficiency because it increases the efficiency of reaching the separated carriers by suppressing recombination during carrier transfer to the electrode.

**Figure 5:**
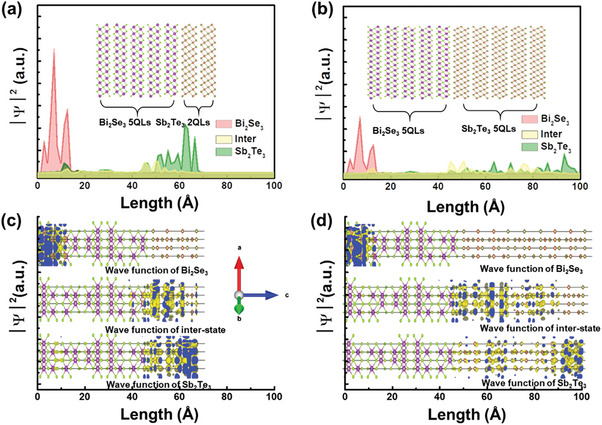
Wave function distribution of Sb_2_Te_3_ a) 2 QL b) 5 QLon Bi_2_Se_3_ 5 QL c), and d) wavefunction of Sb_2_Te_3_/Bi_2_Se_3_ extracted from band structure calculated by density functional theory.

The interfacial band structure of Sb_2_Te_3_/Bi_2_Se_3_ was calculated using the density function theory to confirm changes in the Fermi level related to the surface electronic band structure and the contribution of the surface band to light absorption enhancement, as shown in Figure S5 (Supporting Information). Fort a fixed Bi_2_Se_3_ (5 QLs), the change in the band structure was investigated by varying the thickness of Sb_2_Te_3_ from 2 to 5 QLs. In general, the Fermi level of Bi_2_Se_3_ is located in the conduction band owing to the effect of Se vacancy defects, whereas that of Sb_2_Te_3_ is located in the surface band owing to the *p*‐doping effect of Sb_2_Te_3_. In Sb_2_Te_3_ 2 QL, the Dirac point is located above the Fermi level in Figure S5a (Supporting Information). As Sb_2_Te_3_ thickness increases, the Fermi level and Dirac point overlap (Figure S5b–d, Supporting Information). Moreover, the energy band of the surface state is also increased, indicating that the FCA caused by the surface band structure is increased. The band structure obtained from the density functional theory calculations shows that the Fermi level is modulated by fabricating a junction structure with Bi_2_Se_3_ and Sb_2_Te_3_, which is consistent with the experimental results for OPTP and PPMS. In addition, to analyze the effect of the separation and transport of surface carriers, the wavefunction distribution was extracted from the surface band of each film. **Figure** **5**a,b shows the distribution of the wavefunction extracted from Figure 5c,d, respectively, where red (green) represents the wavefunctions of the Dirac point of Bi_2_Se_3_ (Sb_2_Te_3_), and yellow is the wavefunction extracted from the intermediate state. The wave function of Bi_2_Se_3_ is condensed at the bottom surface regardless of the thickness of Sb_2_Te_3_, as shown in Figure 5a,b. In contrast, the wave function of Sb_2_Te_3_ and the inter‐state are distributed, indicating that, unlike Bi_2_Se_3_ with a 2D surface transport channel, the carriers in Sb_2_Te_3_ move in a 3D space in the Sb_2_Te_3_ region, increasing the probability of photocarrier recombination. The wavefunction distribution and photocurrent characteristics indicate that the observed photocurrent enhancement can occur in the presence of a separate 2D surface transport channel. These findings suggest the potential for significant improvements in the optical properties by developing a system primarily operating on surface channels.

By increasing the amount of light absorption, the photocurrent characteristics can be improved and may also be changed by the electron‐hole separation of the generated photoexcited carriers. Electron–hole separation occurs effectively because of the internal built‐in field in the p–n junction structure.^[^ ^19^, ^30^ ^]^ Ultraviolet photoelectron spectroscopy (UPS) measurements were performed to observe changes in the work function, which revealed the degree of built‐in field (Figure S6, Supporting Information). In the UPS measurements, the sampling depth is below 2 nm. Samples larger than 2 nm showed only the Sb_2_Te_3_ information. Therefore, UPS measurements were performed for 10, 2, 1, and 0 nm thick Sb_2_Te_3_ grown on Bi_2_Se_3_ to track the wavefunction changes for Sb_2_Te_3_, Bi_2_Se_3_, and mixed state, respectively. A change in the work function as a function of Sb_2_Te_3_ thickness is clearly observed. The degree of change in the work function observed using UPS is similar to that measured by XPS. Thus, when a topological insulator‐based p–n junction is formed and the internal field enhances the electron‐hole separation, improving the photocurrent.

**Figure 6:**
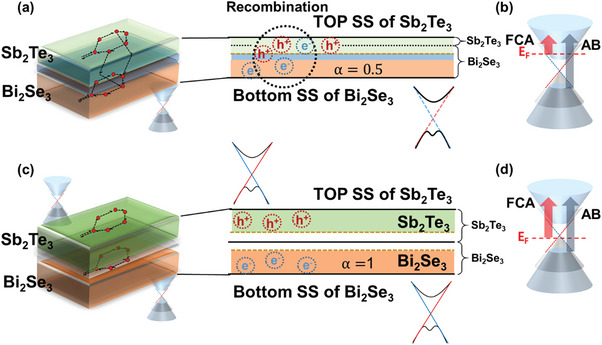
Schematic diagram showing the change in absorption and transport mechanism according to the Fermi level. a,b) Thin Sb_2_Te_3_ and c,d) thick Sb_2_Te_3_ on Bi_2_Se_3._

The mechanism of the enhanced photocurrent generation efficiency in the TPNJ structure can be explained by changes in the absorption and transport mechanisms based on PPMS, UPS, and OPTP. The schematic in **Figure** **6** describes the photocurrent enhancement mechanism. When Sb_2_Te_3_ is thin, absorption bleaching mainly occurs because the Fermi level is located above the Dirac point, FCA decreases, as shown in Figure 6b. However, when Sb_2_Te_3_ is thick, FCA is increased, as shown in Figure 6d. This resulted in a 10% increase in the overall absorption, as shown in Figure 4a. The photoelectrons generated by the irradiated light are then separated through the built‐in field and transported through each surface channel (Top SS of Sb_2_Te_3_ and bottom SS of Bi_2_Se_3_), as shown in Figures 6a,c.

In summary, to compare the photo characteristics of each film with a heterogeneous structure in a one‐chip device, a cross‐pattern device was implemented, and the junction effect was analyzed. The photoresponsivity in the topological p–n junction structure was improved by more than six times compared to that of each material. The absorption change and dynamics were confirmed by controlling the thickness of the Sb_2_Te_3_ grown on Bi_2_Se_3_ to analyze the cause of this improvement. The carrier dynamics and transport characteristics were investigated using PPMS and OPTP results. The photo characteristics were improved by the surface channel with enhanced light absorption and electron–hole separation owing to Fermi level shift.

## 3 Conclusion

This study investigated factors that enhance the optical properties of TPNJ. Experiments and analyses revealed valuable insights into the underlying mechanisms of TPNJs and their potential applications in optoelectronic devices. Through various experiments, it has been confirmed that in addition to bulk effects in TPNJ structures, the surface state and transport channels also play an important role in improving optical properties. This discovery represents a significant breakthrough compared with previous studies that relied solely on band structures. Our study has important implications for developing novel TPNJ‐based optoelectronic devices with improved performances. By understanding the factors influencing the photo characteristics of TPNJs, researchers can design and optimize these devices for specific applications, such as topological insulator‐based photodetectors. Our findings provide exciting possibilities for exploring the potential of TPNJs in optospintronics. The combination of optics and spintronics allows fabricating devices that can manipulate the spin and charge of electrons. The insights gained can be used to develop new materials and fabrication techniques for improved TPNJ‐based device performance and pave the way for realizing next‐generation optoelectronics.

## 4 Experimental Section

### Sample Preparation

Bi_2_Se_3_ 10 QL (1 Quintuple Layer = 0.954 nm) thin films were obtained using the modulated elemental reactants method. Bismuth and Selenium sources were sequentially deposited on a SiO_2_ (300 nm)/Si substrate by thermal evaporation and annealed at 230 ^°^C for 20 min to crystallize the films.^[^ ^45^ ^]^ Then, antimony and tellurium sources were simultaneously deposited on Bi_2_Se_3_ at room temperature. Subsequently, Sb_2_Te_3_ was crystallized by a post‐annealing process.^[^ ^46^ ^]^ All processes were conducted at pressures below 1.2 × 10^−6^ Pa. Details of the growth method are provided in our previous work.^[^ ^20^ ^]^ Ultraviolet photolithography and reactive ion etching were employed to remove the Bi_2_Se_3_ and Sb_2_Te_3_ films, except for the regions covered with a photoresist (AZ GXR 601), to define the channel region. The dimensions of the Bi_2_Se_3_ and Sb_2_Te_3_ channels were 300 × 300 m. The metals for the source – drain electrodes, Ti (25 nm) and Au (25 nm), were deposited by an e‐beam evaporator on the film, leaving only a region of 100 × 100 µm.

### Device Fabrication

The device was fabricated as follows: First, a 10 QL (1 QL = 0.954 nm) Bi_2_Se_3_ thin film was grown on a SiO_2_/Si substrate; a selenium capping layer was then deposited to eliminate atmospheric effects. Subsequently, a Bi_2_Se_3_ channel was fabricated through photolithography and plasma etching; the Sb_2_Te_3_ channel grown on Bi_2_Se_3_ was fabricated by photolithography (Details were added without intermixing with the stacked information) and crystallized by optimizing the annealing temperature without intermixing in the stacked structure. After completing the cross‐pattern, a SiO_2_ capping layer was deposited through sputtering to exclude oxidation.

### Investigation of Photocharacteristics

The photocarriers were generated using a laser diode at a wavelength of 520 nm. The spot size of the laser was less than 10 µm. A 2 V bias voltage was applied, and the laser power was regulated from 200 to 1500 µW. All measurements were performed at room temperature. The photocurrent was measured using a Keithley 2400 source meter.

### Changes in Electronic Structure

A high‐resolution XPS with a monochromatic Al Kα X‐ray source (hν = 1486.7 eV) to investigate changes in the chemical state of Bi_2_Se_3_ and Sb_2_Te_3_ was used. All spectra were calibrated to a carbon reference peak at 284.8 eV. UPS measurements with an ultraviolet light source (He I, 21.22 eV) tracked changes in the work function due to surface oxidation on Bi_2_Se_3_. A sample bias voltage of −10 V was applied to obtain the secondary electron cutoff region.

### Structure Analysis

The Raman spectra of Bi_2_Se_3_ were obtained by micro‐Raman spectroscopy (Horiba Lab Ram ARAMIS) using a 532 nm‐wavelength Nd:YAG laser, 100× objective, and 2400 grooves mm^−1^ grating. We calibrated the spectra to a silicon peak of 520 cm^−1^.

### Electrical Measurement

The electrical properties of Bi_2_Se_3_ were measured with a four‐point probe using a standard Hall bar configuration of size 20 µm fabricated using photolithography. MR measurements were conducted using magnetic fields of up to 3.5 T applied perpendicular to the films over 2–300 K using PPMS.

### Thz Spectroscopy

A Ti: sapphire regenerative amplifier laser system with a repetition rate of 1 kHz and a center wavelength of 800 nm was used for the THz‐TDS and pump‐probe measurements. The output pulse of the regenerative amplifier was divided into two parts. The first part was used to generate and detect THz probe pulses via the optical rectification by using the 10 × 10 mm〈110〉ZnTe crystal. The transmitted THz pulse was detected by the electro‐optic sampling method. The other part of the laser pulses was delivered to a sample for optical pumping with a time difference via an optical delay line for pump‐probe measurements.The signal current was recorded via a lock‐in amplifier (Stanford Research System, SR3830) to remove any residual pump beam. All samples used in this work were loaded onto the holder with a 3 mm hole to confine the measuring spot. All THz experiments were performed in a sealed box under dry air at room temperature.

### DFT Calculation

DFT calculations were performed using the Vienna ab initio simulation package (VASP) with the GGA‐PBE sol functional.^[^ ^47^, ^48^, ^49^ ^]^ Initially, the geometry of the Bi_2_Se_3_ and Sb_2_Te_3_ unit cells was optimized using the RMM‐DIIS algorithm until the 0.02 eV/Å convergence criterion was satisfied. To ensure a k‐spacing of less than 0.2/Å, a 9 × 9 × 9 grid of k‐points was selected, with a cutoff energy set to 500 eV. Based on the optimized unit cell, models with 5 QLs of Bi_2_Se_3_ interfaced with 1–5 QLs of Sb_2_Te_3_ were created, and geometry optimization was carried out until the 0.05 eV Å^−1^ convergence criterion was met. Subsequently, electronic structure calculations of the interface models were performed while considering spin‐orbit coupling (SOC). For the interface model calculations, a 9 × 9 × 1 grid of *k*‐points and a cutoff energy of 500 eV were utilized.

## Conflict of Interest

The authors declare no conflict of interest.

## Supporting information

Supporting Information

## Data Availability

The data that support the findings of this study are available on request from the corresponding author. The data are not publicly available due to privacy or ethical restrictions.
