# Supplementary material for: Enhanced Photocharacteristics by Fermi Level Modulating in Sb2Te3/Bi2Se3 Topological Insulator p–n Junction
Source: Adv Sci (Weinh). 2023 Dec 31;11(11):2307509. doi: 10.1002/advs.202307509 (PMC10953576; doi:10.1002/advs.202307509)
Supplement: Supplementary file 1 — Supporting Information [file ADVS-11-2307509-s001.pdf]

ADVANCED SCIENCE
Open Access

Supporting Information

for *Adv. Sci.*, DOI 10.1002/advs.202307509

Enhanced Photocharacteristics by Fermi Level Modulating in Sb_2Te_3/Bi_2Se_3 Topological Insulator p–n Junction

*Seok-Bo Hong, Dajung Kim, Jonghoon Kim, Jaehan Park, Seungwon Rho, Jaeseok Huh, Youngmin Lee, Kwangsik Jeong and Mann-Ho Cho**

Supporting Information

Enhanced photo-characteristics by fermi level modulating in Sb_2Te_3/Bi_2Se_3 Topological PN junction

Seok-Bo Hong[1], Dajung Kim[1], Jonghoon Kim[1], Jaehan Park[1],Seungwon Rho[1], Jaeseok Huh[1], Youngmin Lee[1], Kwangsik Jeong[3*] and Mann-Ho Cho[1,2*]

1. Device fabrication

The characteristics of a single thin film and the junction structure could be compared under the same conditions according to the source-drain metal contact. A cross pattern was devised, as shown in Figure S1, to confirm only the junction effect at the interfaces, excluding external influences. The device was fabricated as follows: First, the SiO_2 layer on the SiO_2/Si substrate was etched using photolithography to deposit Bi_2Se_3 with an etching depth of 10 nm corresponding to approximately 10 QL (1 QL = 0.954 nm) Bi_2Se_3 thin film. The Bi_2Se_3layer was then grown on a SiO_2/Si substrate, and a selenium capping layer was deposited to eliminate atmospheric effects. Photolithography and plasma etching were used to fabricate the Bi_2Se_3 channel, and a Sb_2Te_3 channel was grown on the Bi_2Se_3 channel using photolithography. The annealing temperature was optimized to prevent intermixing in the stacked structure and crystallize the Sb_2Te_3 layer above the Bi_2Se_3 layer. After completing the cross pattern, a SiO_2 capping layer was deposited using sputtering to exclude oxidation. This process effectively prevented height differences during subsequent Sb_2Te_3 deposition, creating a high-quality topological insulator junction structure.

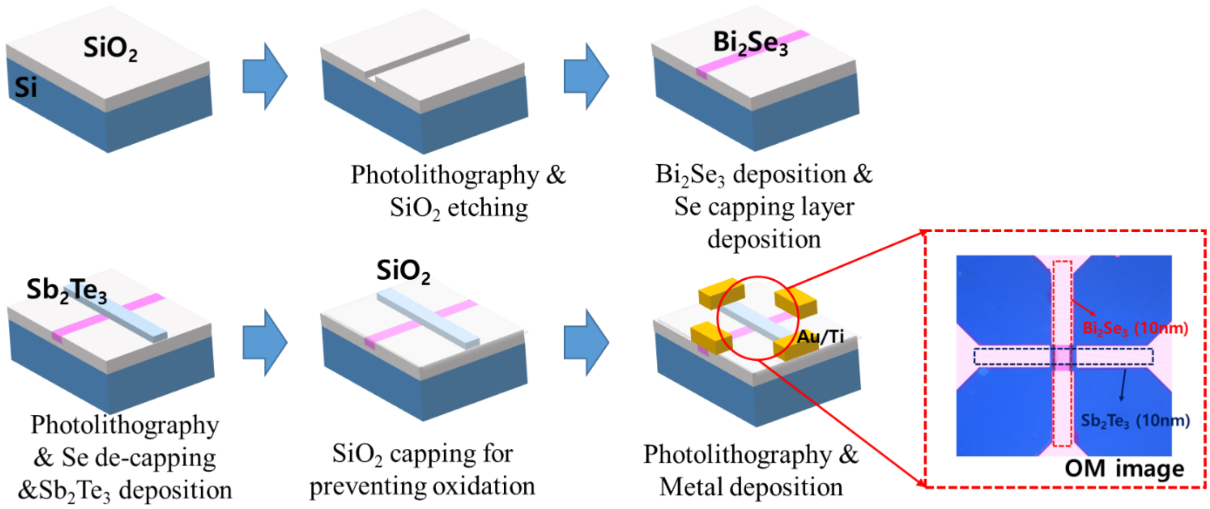

Figure S1. Schematic illustration of the device fabrication process for the Sb_2Te_3/Bi_2Se_3 topological insulator junction

2. Interface analysis

The STEM analysis confirmed the formation of a well-defined interface. A comparison of the intensity in the line profile analysis enabled the distinction between the cases with and without mixed layers. In Figure S2a, where the interface is well separated, the boundary is clearly visible in the line profile, as shown in Figure S2c. However, an intermediate layer appears for S2b, where the interface is mixed, as shown in S2d. The intermediate layer consists of two layers: the first layer is the Se-rich SbSeTe, and the second layer is the Sb-rich SbSeTe. This was confirmed through intensity comparisons during line profile analysis.

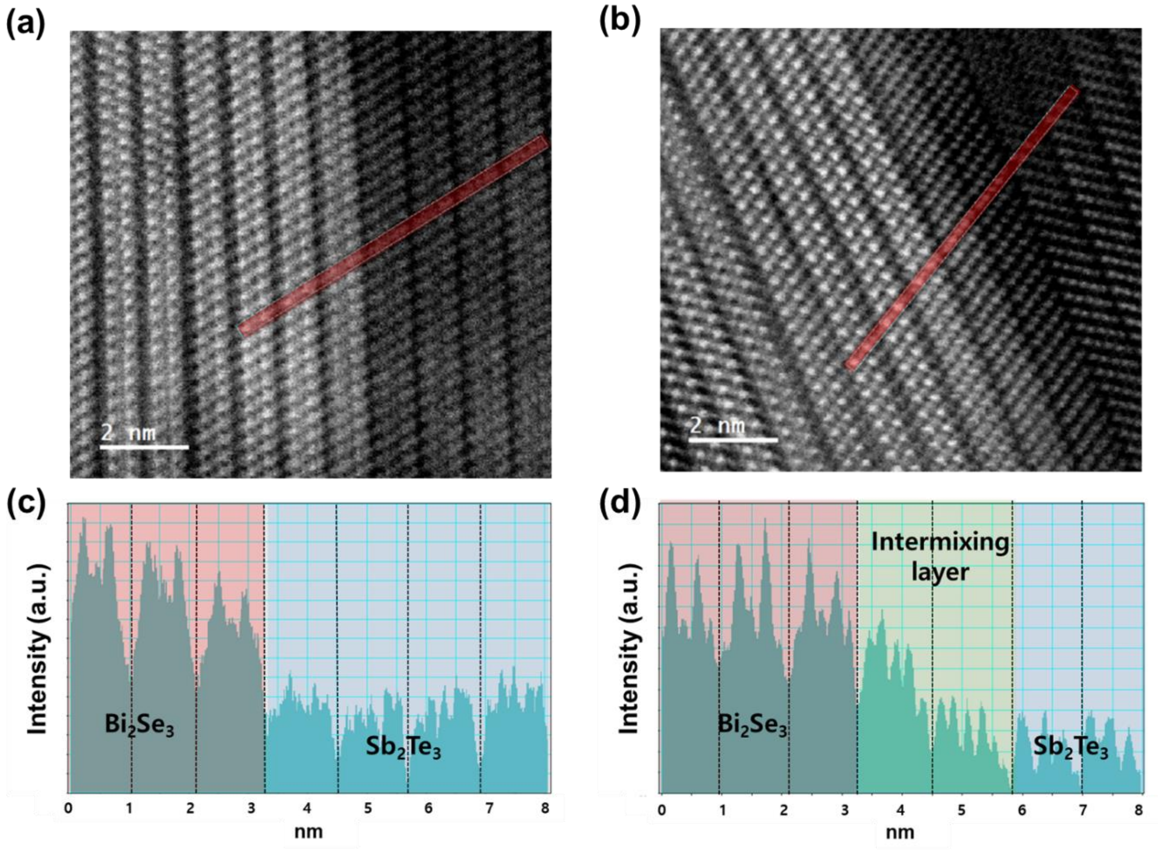

Figure S2. STEM images and corresponding line profiles of the interfaces between Bi_2Se_3 and Sb_2Te_3 layers. The line profiles reveal a clear distinction between the layers with a well-defined interface (Figure S2a,c) and those with intermixing at the interface (Figure S2b,d).

Although it was confirmed that no intermixing layer exist at the interface, we confirmed changes in the chemical bonding state at the interface, influencing the junction effect through angle-resolved X-ray photoelectron spectroscopy (AR-XPS). AR-XPS measurements can

confirm changes in the chemical bonding state at the Bi_2Se_3 and Sb_2Te_3 interfaces because the information according to the depth of the sample appeared depending on the angle at which the X-rays were incident on the sample. As the X-rays were incident vertically, deeper depth information appeared, and information on Bi_2Se_3 and Sb_2Te_3 was mixed. When the X-rays were incident closer to 0°, information on Sb_2Te_3, which is surface information, appeared dominantly. The chemical bonding states at the interfaces were confirmed through angle-dependent changes in the binding energy. The core-level XPS spectra of Sb_2Te_3/Bi_2Se_3 were obtained as a function of the incident angle from 0 to 85°, as shown in Figure S3a-d. The peak positions of all spectra were calibrated using the reference carbon peak at 284.8 eV; each peak was deconvoluted to observe the chemical bonding state clearly. The Bi core-level spectra of Bi_2Se_3/Sb_2Te_3 show the characteristic doublet representing Bi $4f_{5/2}$ and $4f_{7/2}$ orbitals, with peaks at 163.3 eV and 158 eV in Figure S3a, respectively, which is consistent with previously reported data. The Se core-level spectra also show doublet peaks of Se $3d_{3/2}$ and $3d_{5/2}$ at 54.25 and 53.4 eV, respectively, in Figure S3b. The Sb core-level (538.5 eV for Sb $3d_{3/2}$ and 529.2 eV for $3d_{5/2}$) and Te core-level spectra (583.1 eV for Te $3d_{3/2}$ and 572.7 eV for $3d_{5/2}$,) were also consistent with previous reports in Figure S3c-d. It was confirmed that no shift in the binding energy occurred due to the angle change or new chemical bonding. Therefore, the intermixing did not occur between the two materials at the interface, and the junction structure was effectively fabricated through heat treatment control. As shown in Figure S3e, the valence band maximum shifted by approximately 0.26 eV. That is, there was no change in the chemical bonding at the interfaces, but band bending, which improved electron-hole separation, occurred. However, when annealed at 180°, a binding-energy shift occurred in the Bi and Se atoms when using a low-angle incident beam, as shown in Figure S4. Additionally, the change in VBM decreased from 0.26 to 0.11 eV compared to when a clean interface was formed through annealing at 170°. This suppresses electron-hole separation and reduces the optoelectronic properties. The XRD, Raman, STEM, and AR-XPS results confirmed that a Sb_2Te_3/Bi_2Se_3 topological p-n junction structure was effectively formed without intermixing by controlling the annealing conditions.

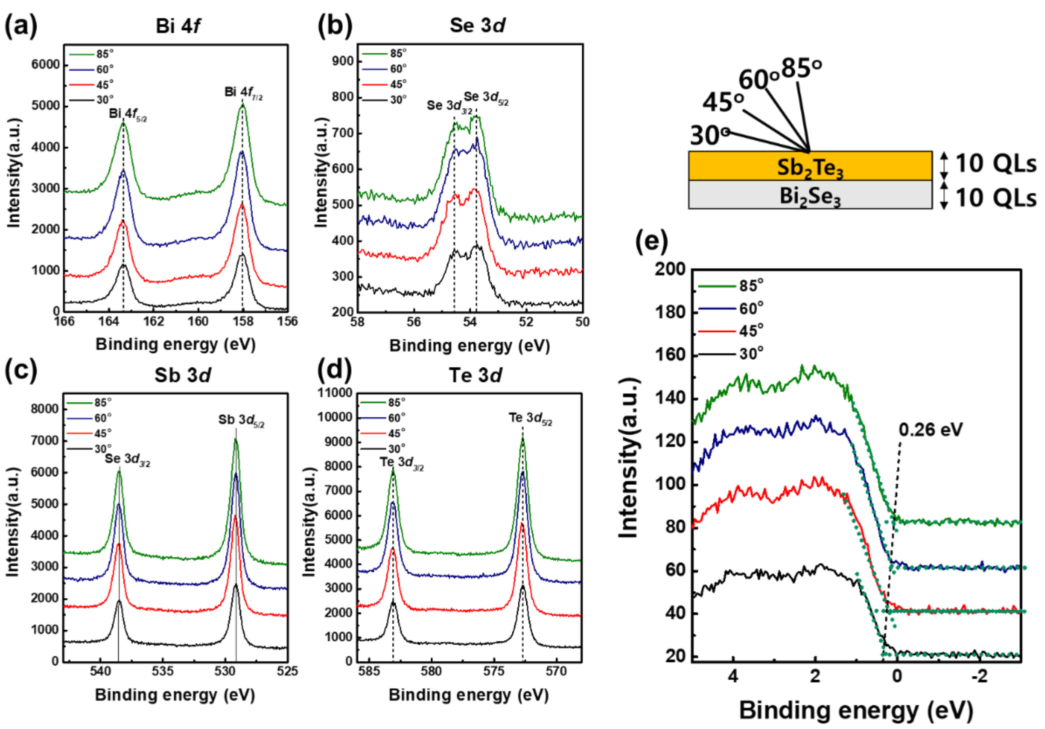

Figure S3. Results of angle-resolved X-ray photoelectron spectroscopy in Sb_2Te_3/Bi_2Se_3 at 170 °C annealing temperature (a) Bi 4*f*, (b) Se 3*d*, (c) Sb 3*d*, (d) Te 3*d*, (e) change in valence band maximum (VBM) depending on the angle of incidence. The schematic shows the angle of incidence

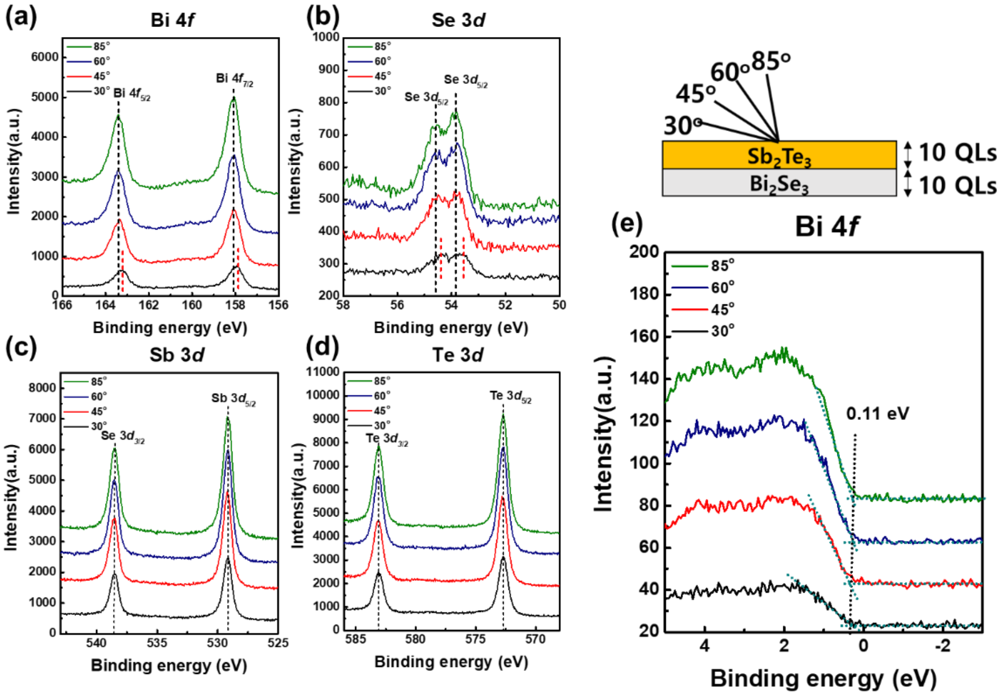

Figure S4. Results of angle-resolved X-ray photoelectron spectroscopy in Sb_2Te_3/Bi_2Se_3 at 180 °C annealing temperature: (a) Bi 4*f*, (b) Se 3*d*, (c) Sb 3*d*, (d) Te 3*d*, and (e) change in VBM depending on the angle of incidence.

Density functional theory (DFT) was used to investigate the interfacial band structure of Sb_2Te_3/Bi_2Se_3 and its relationship to changes in the Fermi level and the light absorption contribution of the surface band, as shown in Figure S5. We observed a change in the band structure by fixing Bi_2Se_3 thickness at 5 QLs and varying Sb_2Te_3 thickness from 2 to 5 QLs. The yellow curve represents the band structure of Sb_2Te_3, and the blue curve represents that of Bi_2Se_3, with the interstate shown as a red area. When Sb_2Te_3 thickness is 2 QLs, the interstate is below the Fermi level, as shown in Figure S5a. Generally, the Fermi level of Bi_2Se_3 is in the conduction band owing to Se vacancy defects, whereas the interstate in Bi_2Se_3/Sb_2Te_3 is located above the Fermi level. As the Sb_2Te_3 thickness increases, the inter-state move closer to the Fermi level, eventually intersecting it, as shown in Figure S5-d. The Fermi level then shifts to the Dirac point of Sb_2Te_3, which increases the energy band of the surface state, and consequently, enhances surface absorption (FCA). Our DFT calculations confirm that the Fermi level can be modulated by constructing a junction structure of Bi_2Se_3 and Sb_2Te_3, which is consistent with our experimental observations using OPTP and PPMS.

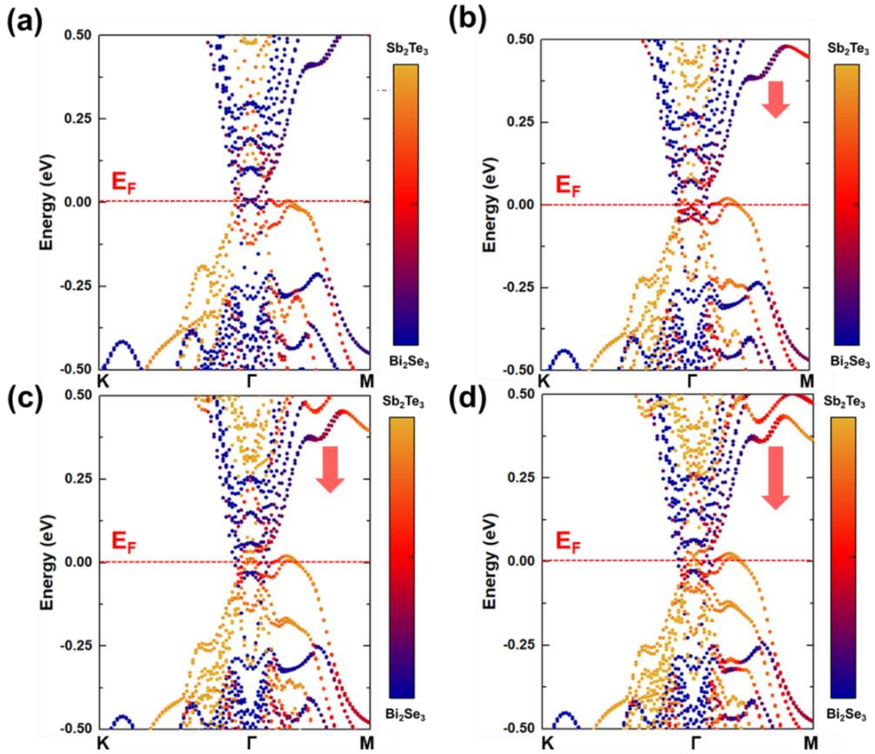

Figure S5. Band structure from DFT calculation for Sb_2Te_3/Bi_2Se_3 junctions with different Sb_2Te_3 thicknesses: (a) 2 QLs, (b) 3 QLs, (c) 4 QLs, and (d) 5 QLs on 5 QLs of Bi_2Se_3.

Ultraviolet photoelectron spectroscopy (UPS) measurements were conducted to numerically confirm the Fermi level shift and the influence of band bending. The work function of pristine Bi_2Se_3 is measured at 4.76 eV. As Sb_2Te_3 was grown on the Bi_2Se_3, the work function progressively increases, indicating the movement of the Fermi level towards the valence band. When Sb_2Te_3 reaches 1 QLs in thickness, the work function is measured at 4.86 eV, and it further increases to 4.90 eV with 2 QLs of Sb_2Te_3, indicating a gradual lowering of the Fermi level. Ultimately, when Sb_2Te_3 reaches a thickness of 10 QLs, the work function stabilizes at approximately 4.92 eV. Considering Sb_2Te_3's approximate band gap of 0.3 eV (*Scientific Reports* **2015**, 5(1)), the 0.16 eV change in work function indicates that the Fermi level is stably positioned within the bulk band gap, as illustrated in Figure S7. Additionally, the approximately 0.16 eV difference in work function between Bi_2Se_3 and Sb_2Te_3 implies the formation of a built-in field in the Sb_2Te_3/Bi_2Se_3 junction structure, which can lead to electron-hole separation, thereby enhancing optical properties

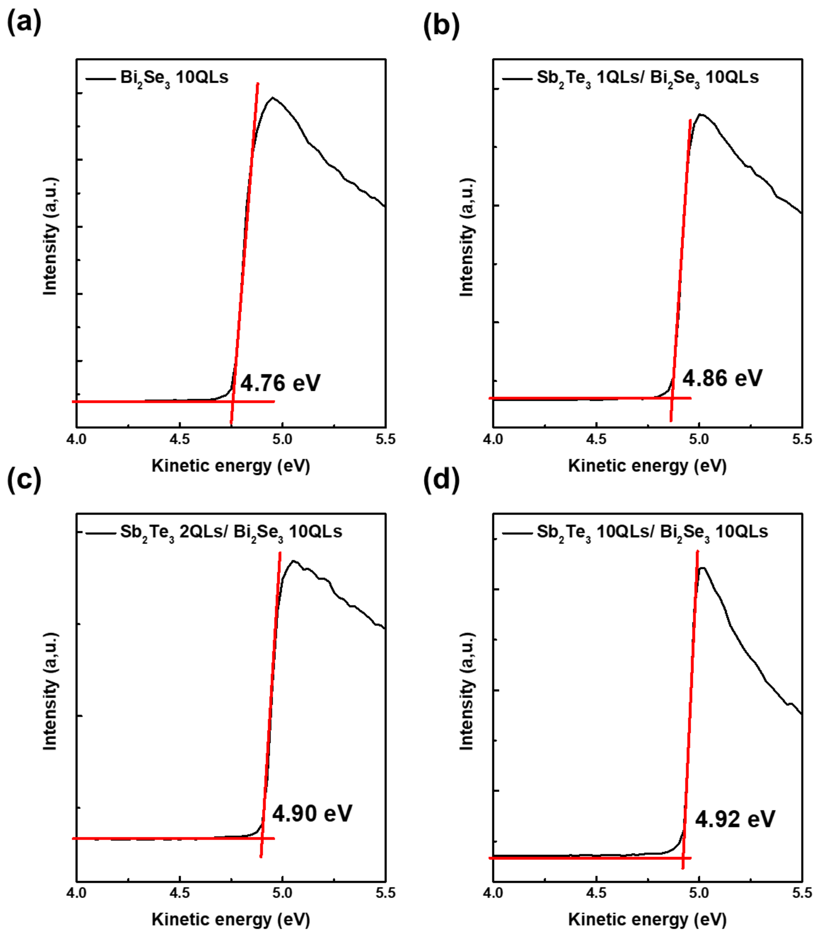

Figure S6. Ultraviolet photoelectron spectroscopy results of (a) Bi_2Se_3 10 QLs, (b) Sb_2Te_3 1 QLs /Bi_2Se_3 10 QLs, (c) Sb_2Te_3 2 QLs/Bi_2Se_3 10 QLs (d) Sb_2Te_3 10 QLs/Bi_2Se_3 10 QLs"

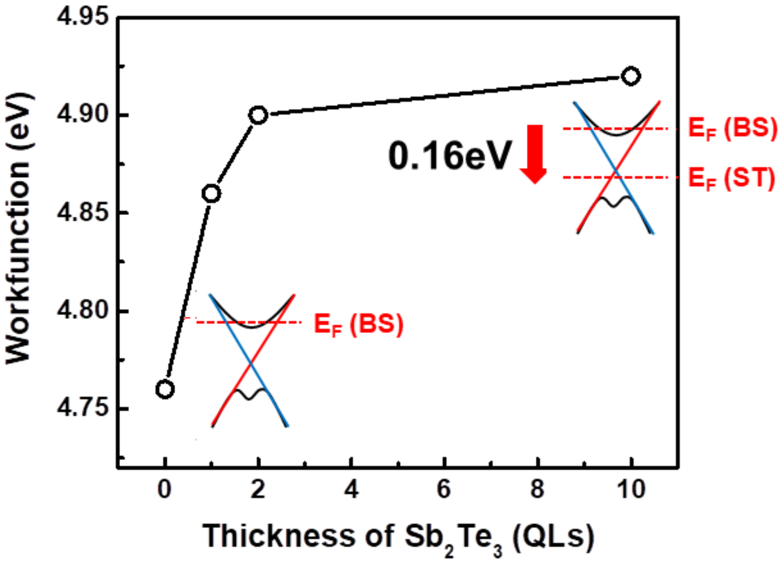

Figure S7. The changes in workfunction as a function of Sb_2Te_3 thickness grown on Bi_2Se_3 10 QLs

The Hikami-Larkin-Nagaoka (HLN) equation that relates the change of magneto-conductivity ($\Delta\sigma(H)$) to various physical parameters is expressed as follows:[40]

$$\Delta\sigma(H) = \sigma(H) - \sigma(0) = \frac{\alpha e^2}{\pi h}\left[\ln\left(\frac{B\varphi}{H}\right) - \Psi\left(\frac{1}{2} + \frac{B\varphi}{H}\right)\right] \text{ ------ (1)}$$

where Ψ is the digamma function, e is the electronic charge, and h is Planck's constant. The characteristic magnetic field is represented by $B\varphi$, which is determined by the phase coherence length ($l\varphi$) and coefficient (α) that characterize the localization type. The applied magnetic field is denoted as H. In topological insulators, α is related to the surface channel and equals 0.5 when there is a single surface channel. Figure S7a shows that the α = 0.74 for Bi_2Se_3 as the top and bottom surface channels partially hybridize each other. In Sb_2Te_3 with 2 QLs deposited, the top surface channel of Sb_2Te_3 does not exist independently and hybridizes with the bottom surface channel of Bi_2Se_3. Since there is no topologically different surface channel at the interface between Bi_2Se_3/Sb_2Te_3, there is no surface channel. As Sb_2Te_3 thickness increases and an independent surface channel is formed on the top surface, α becomes 0.5; the overall α value becomes 1 due to the top and bottom surface channel of Bi_2Se_3, as shown in Figure S7d.

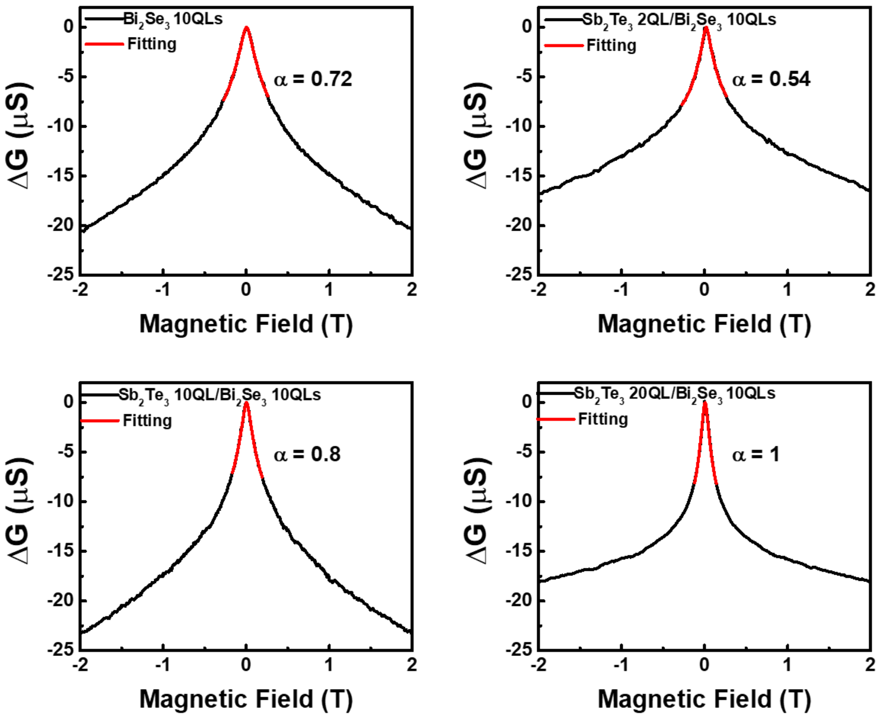

Figure S8. PPMS fitting results based on the thickness of Sb_2Te_3 grown on Bi_2Se_3. (a) Bi_2Se_3 10 QLs (b) Sb_2Te_3 2 QLs/ $Bi_2Se_3$10 QLs (c) Sb_2Te_3 10 QLs/ $Bi_2Se_3$10 QLs (d) Sb_2Te_3 20 QLs/ $Bi_2Se_3$10 QLs

3. Photo-characteristics of Sb_2Te_3/Bi_2Se_3

The photocurrent results were analyzed as a function of the incident power, wavelength, and thickness. The photocurrent was found to increase with increasing power, but the dependence on the wavelength and thickness varied. As the thickness increased to 10 QLs, the photocurrent initially increased and then decreased. Furthermore, as the thickness increased, the difference in the photocurrent intensity as a function of power became more distinct, indicating a clear dependence on power. In addition, when fitted with a power law, the theta value increases, indicating an increase in the electron-hole separation.

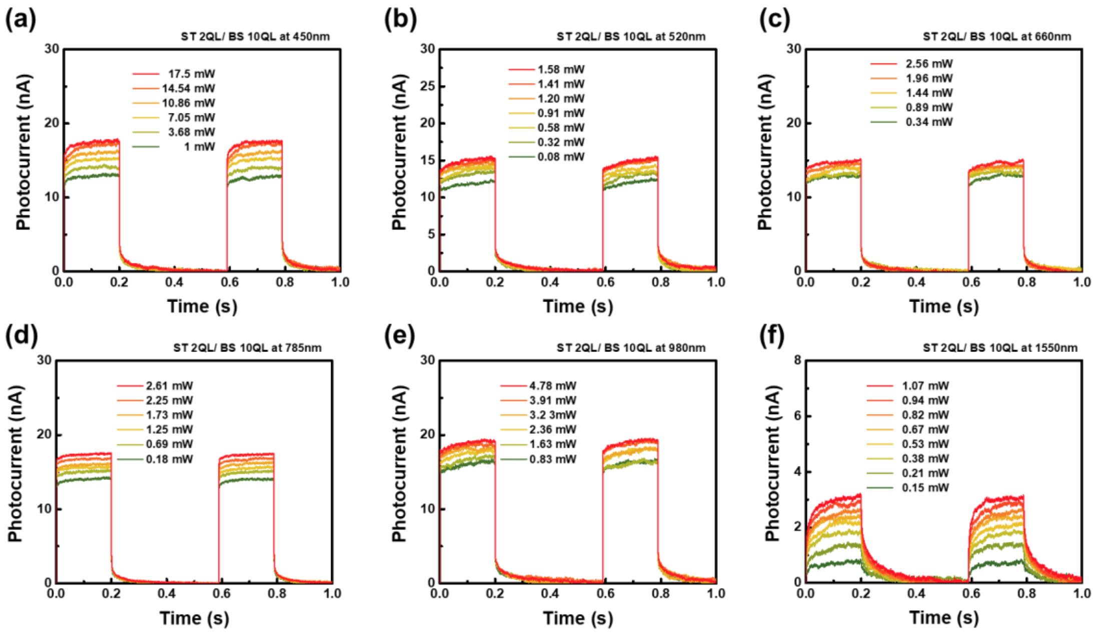

Figure S9. Photo-characteristics of Sb_2Te_3 2 QLs / Bi_2Se_3 10 QLs depending on the wavelength as a function of power: (a) 450 nm (b) 520 nm (c) 660 nm (d) 785 nm (e) 980 nm (f) 1550 nm

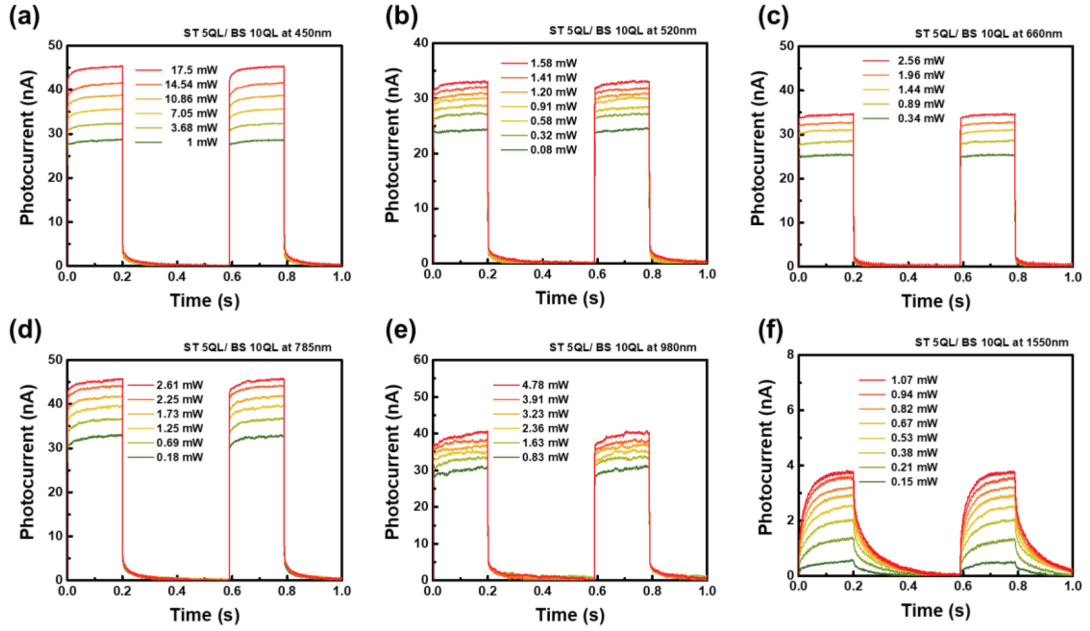

Figure S10. Photo-characteristics of Sb_2Te_3 5 QLs / Bi_2Se_3 10 QLs depending on the wavelength as a function of power: (a) 450 nm (b) 520 nm (c) 660 nm (d) 785 nm (e) 980 nm (f) 1550 nm

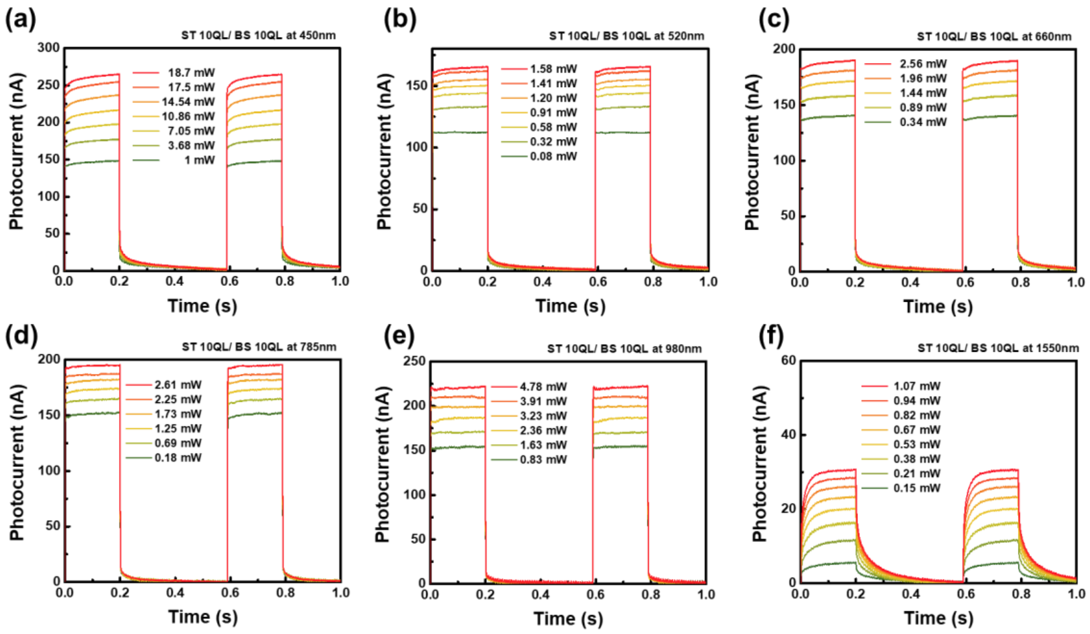

Figure S11. Photo-characteristics of Sb_2Te_3 10 QLs / Bi_2Se_3 10 QLs depending on the wavelength as a function of power: (a) 450 nm (b) 520 nm (c) 660 nm (d) 785 nm (e) 980 nm (f) 1550 nm

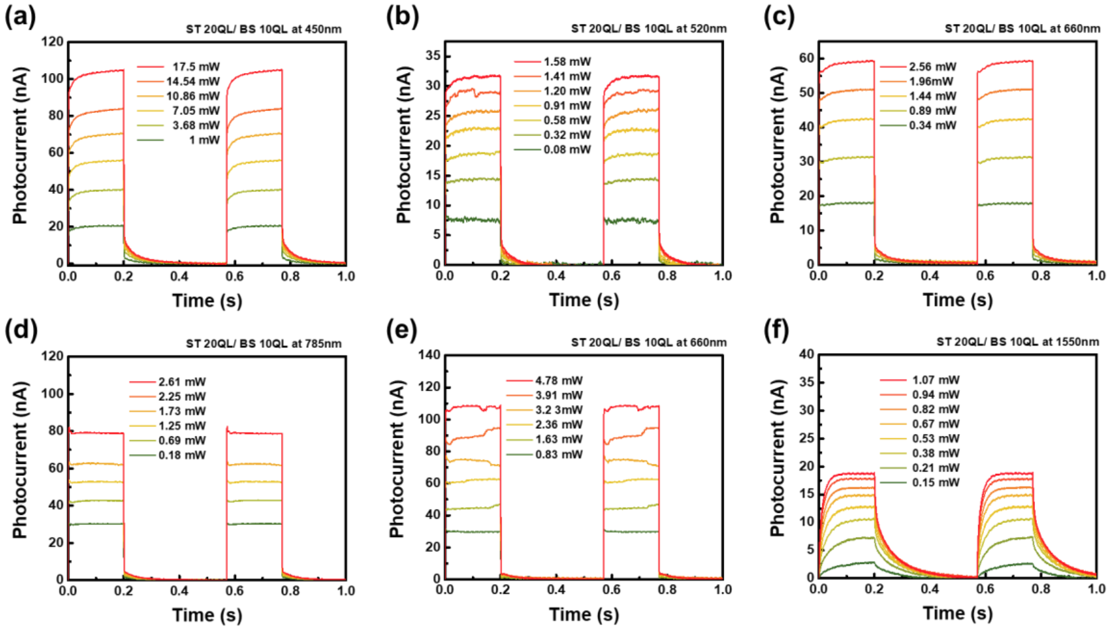

Figure S12. Photo-characteristics of Sb_2Te_3 20 QLs / Bi_2Se_3 10 QLs depending on the wavelength as a function of power: (a) 450 nm (b) 520 nm (c) 660 nm (d) 785 nm (e) 980 nm (f) 1550 nm

4. Role of defect in TPNJ structure

Densitty functional theroy (DFT) calculations were performed on each model, namely Bi_2Se_3, Sb_2Te_3, and Bi_2Se_3/Sb_2Te_3, to assess the influence of defects. Our focus was on Se and Te vacancies while prioritsing energetically stable defects in the formation process. Prior studies (Superlattices and Microstructures 2018, 120, 48-53, and PRL 2012, 108, 066809) were consulted as guides for our work. A 2 × 2 × 1 supercells were constructed based on the optimized structures of 5 QLs Bi_2Se_3, 5 QLs Sb_2Te_3, and 5 QLs Sb_2Te_3/Bi_2Se_3 5 QLs, as well as 2 QLs Sb_2Te_3/Bi_2Se_3 5 QLs. Supercell calculations employed a 3 × 3 × 1 grid of k-points and 500 eV cut-off energy. Geometry optimizations were performed for supercells containing defects until 0.05 eV/Å condition was satisfied. Subsequently, electronic structures of the supercells were calculated, considering spin-orbit coupling (SOC). Band structures for supercells are unfolded based on the effective band structures (EBS) method, as referenced *Phys. Rev. B* 85, 085201 (**2012**).

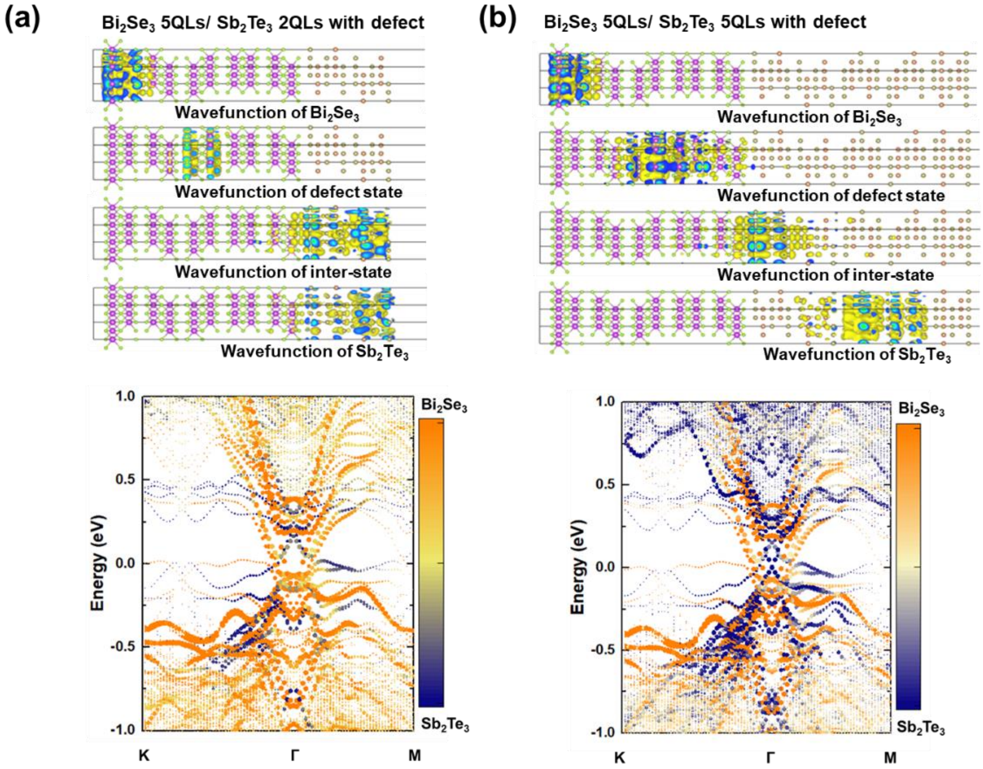

Figure S13. The band structure of (a) Sb_2Te_3 2 QLs and (b) Sb_2Te_3 5 QLs on Bi_2Se_3 5 QLs with a defect state. The upper panels illustrate the spatial wavefunction distribution extracted from the band structure

The band structure was recalculated to consider the effects of defects, and the wave function was extracted, as depicted in Figure S13. In panels (a) and (b), colors represent the contributions of Bi_2Se_3

and Sb_2Te_3, respectively. The red color indicates the contribution from Sb_2Te_3, while the blue color represents the contribution from Bi_2Se_3. Point size signifies the quantity of states in the effective band. The isosurface level has been fixed at $5x10^{-12}$ e/Å^3 because variations in electron density can occur based on the isosurface level value. The band index information for each wavefunction is provided in Figure S14.

As a result, we identified deformed wavefunctions mainly located in the Bi_2Se_3 region due to defect states. This suggests that the defect state has little influence on the interaction between the wavefunction of Bi_2Se_3 and Sb_2Te_3. Furthermore, despite the reduction in wavefunction distribution due to defects, it is noteworthy that in thicker regions, similar to the initial analysis, the inter-state and Sb_2Te_3 wavefunctions remain separate and distinct. Therefore, even when considering the influence of defects, the absorption contributions of the surface states and channel separation continue to occur, indicating an enhancement in optical properties."

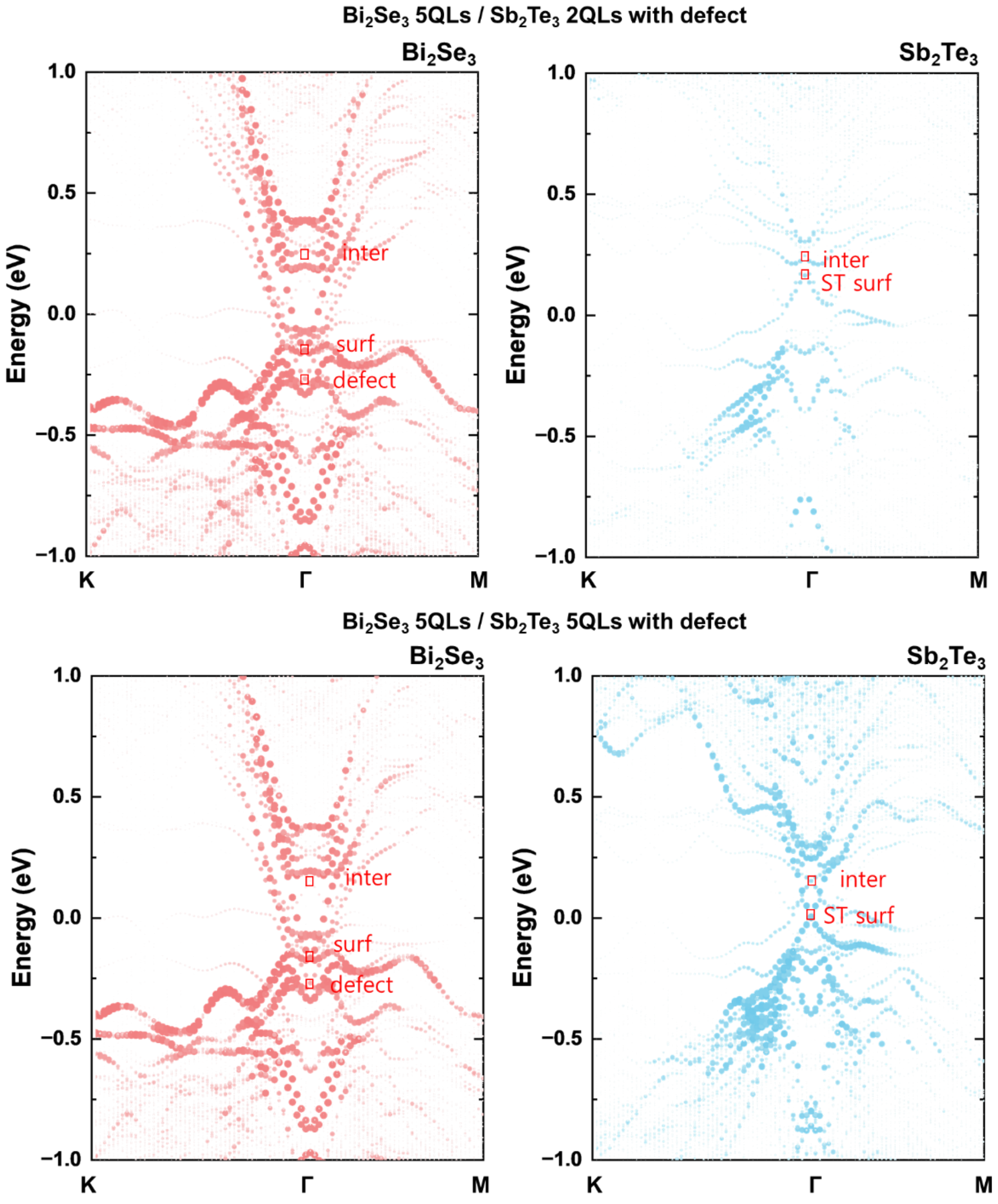

Figure S14. Information regarding the band index of the wavefunctions.

5. Temperature dependent resistivity curve according to the thickness of Sb_2Te_3 grown on Bi_2Se_3

We performed Resistivity-Temperature (R-T) curve measurements for Sb_2Te_3 grown on Bi_2Se_3 at varying thicknesses and observed insulating behavior for the Sb_2Te_3 10 QLs/Bi_2Se_3 10 QLs sample, as shown in Figure S14. According to previous research, when the Fermi level is located within the bulk band gap, the material exhibits insulating behavior, whereas when it transitions into the bulk band, it demonstrates metallic behavior. As can be observed

in Figure S14, for Sb_2Te_3 with 5 QLs and 10 QLs grown on Bi_2Se_3 with 10 QLs, it exhibits insulating behavior, and as the thickness of Sb_2Te_3 increases, it shows metallic behavior. This suggests that the Fermi level is shifting with the thickness of Sb_2Te_3, and for the prominently discussed sample in the main text, Sb_2Te_3 10 QLs/Bi_2Se_3 10 QLs, the Fermi level is positioned within the bulk band gap

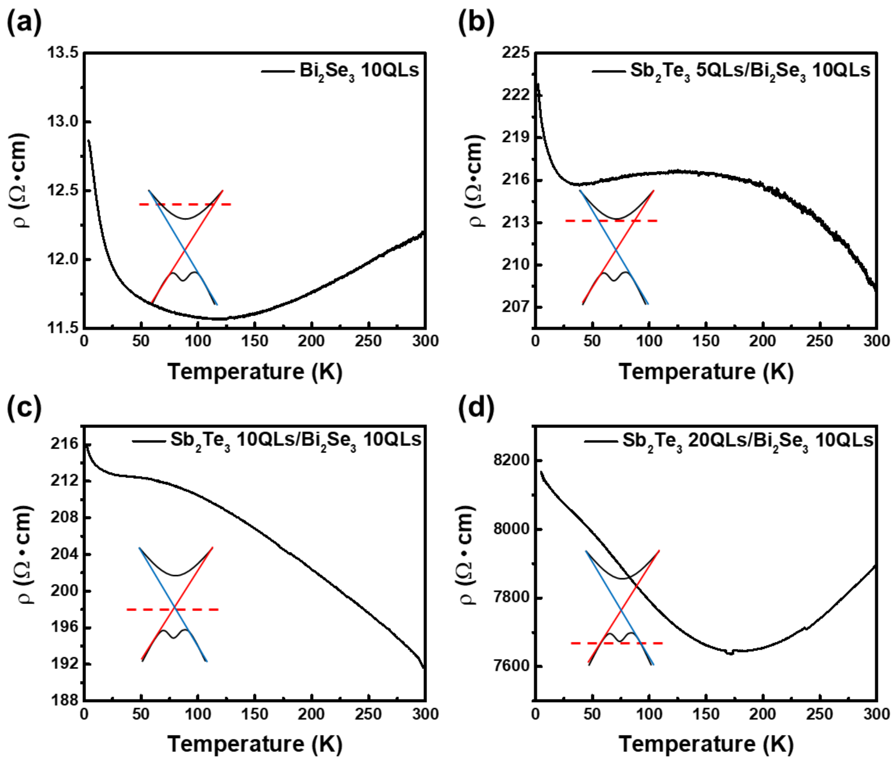

Figure S15. Resistivity-Temperature curve according to the Sb_2Te_3 thickness growing on Bi_2Se_3. (a) Bi_2Se_3 10 QLs (b) Sb_2Te_3 5 QLs/ Bi_2Se_3 10 QLs (c) Sb_2Te_3 10 QLs/ Bi_2Se_3 10 QLs (d) Sb_2Te_3 20 QLs/ Bi_2Se_3 10 QLs

6. Comparison of Responsivity in Photodetectors Based on Topological Insulators

In Table S1, we provide Figure of Merits for TPNJ structures along with references to previously reported studies based on topological insulators

Table S1. Figure of Merits for the photodetectors Based on Topological Insulators

Materials and devices	Wavelength (nm)	Photocurrent (uA)	Responsivity (AW^{-1})	Response time (us)	Ref
Sb_2Te_3/Bi_2Se_3	520	0.34	0.34	300	Our work
Bi_2Se_3	520	0.06	0.06	400	Our work
Sb_2Te_3	520	0.004	0.004	300000	Our work
$SnTe/Bi_2Se_3$	1550	74.9	0.146	6.9	29
Sb_2Te_3	635	22.5	0.00136	14220	49

Table S1 reveals that the reported responsivity in the previously studied TPNJ structures is approximately 0.146 A/W, which is lower than the achieved 0.34 A/W in our study. [29] Furthermore, the optical characteristics of the TPNJ structure exhibit improvements co individual topological insulators. In photodetectors, responsivity can vary significantly depending on factors such as light intensity, wavelength, voltage, and more. [49] Therefore, evaluating the significance of research based solely on performance metrics can be challenging. Nonetheless, our research remains significant as it allows for a specific and direct comparison of the characteristics of each channel within a single device. This enables us to quantitatively assess the improvement achieved in the Bi_2Se_3/Sb_2Te_3 structure compared to individual Bi_2Se_3 and Sb_2Te_3 materials. As a result, we can confirm that the responsivity has improved by more than six-fold in our study when compared to the individual materials. Our research primarily acknowledges the potential of the TPNJ structure, as well as serves as a pioneering investigation with the significance of understanding the contribution of topological surface states in TPNJ structures, which can find applications in the field of opto-spintronics.

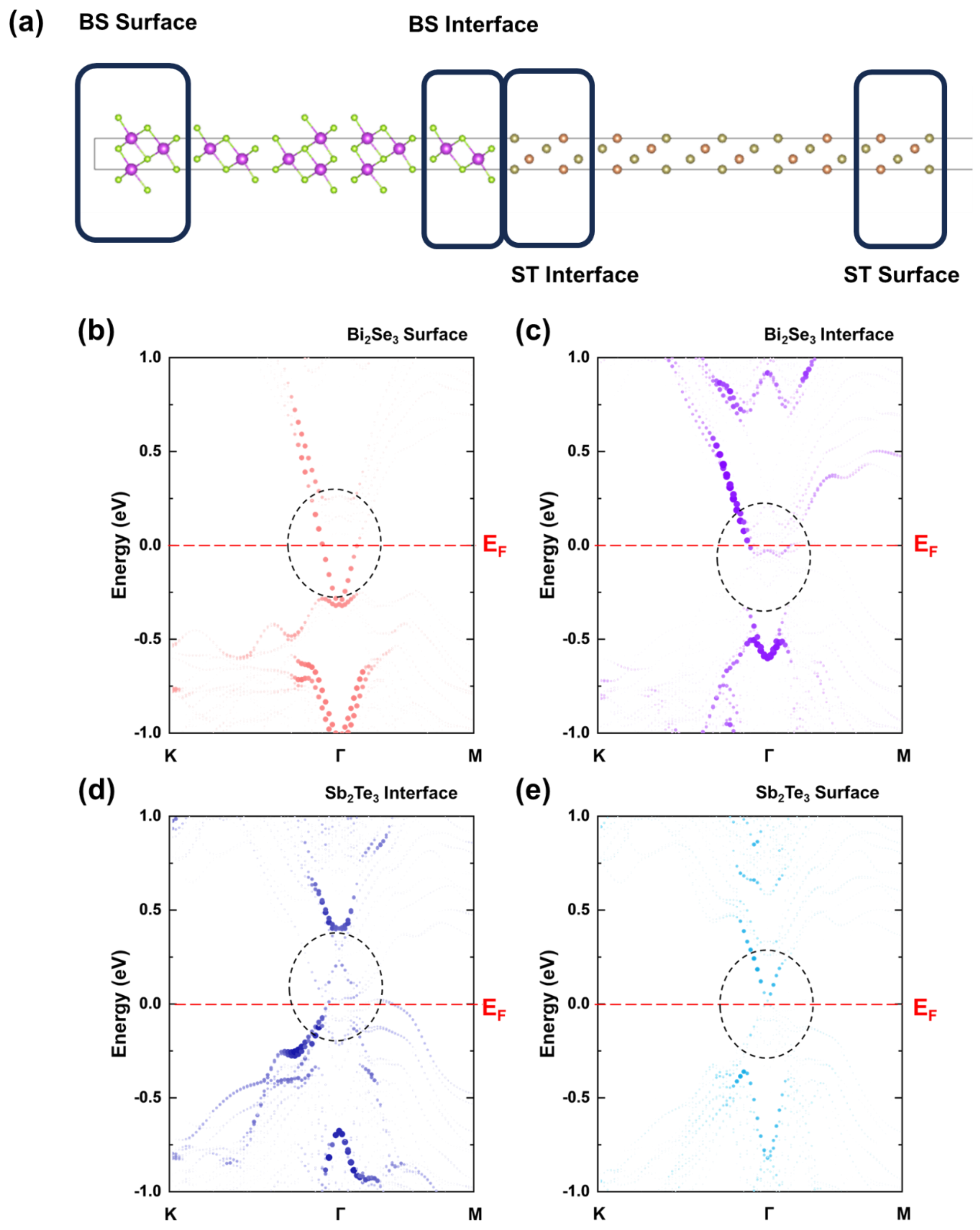

Figure S16. (a) Schematic image of the calculated band structure for the specific region. The extracted band structures at the positions indicated in Figure S14 (a) for Bi_2Se_3 5QLs / Sb_2Te_3 5QLs. (b) Bi_2Se_3 surface, (c) Bi_2Se_3 interface, (d) Sb_2Te_3 interface, and (e) Sb_2Te_3 surface.

To delve more deeply into the information of not only the bulk but also the surfaces and interfaces, additional calculations of the band structure for each surface and interface have been conducted and incorporated as depicted in Figure S14a. This allows for a more detailed examination and separation of the band structures at the surfaces and interfaces. Based on the band structure calculations at the

positions indicated in Figure S14a, the results are depicted in Figures S14b-d. The Fermi level is positioned within the surface bands on the bottom surface of Bi_2Se_3 and the top surface of Sb_2Te_3, respectively. Based on these results, it is evident that the increased absorption efficiency may be attributed to the absorption effects of the surface bands according to the Fermi level's position.
